# Designing Novel Strategy to Produce Active Nanohybrids in Sunlight for Purification of Water Based on Inorganic Nanolayers, Magnetic Nanocomposites and Organic Species

**DOI:** 10.3390/molecules27123673

**Published:** 2022-06-07

**Authors:** Osama Saber, Mostafa Osama, Nagih M. Shaalan, Aya Osama, Adil Alshoaibi, Doaa Osama

**Affiliations:** 1 Al Bilad Bank Scholarly Chair for Food Security in Saudi Arabia, The Deanship of Scientific Research, The Vice Presidency for Graduate Studies and Scientific Research, King Faisal University, P.O. Box 400, Al-Ahsa 31982, Saudi Arabia; 214110595@student.kfu.edu.sa (M.O.); nmohammed@kfu.edu.sa (N.M.S.); 217044956@student.kfu.edu.sa (A.O.); adshoaibi@kfu.edu.sa (A.A.); 221445253@student.kfu.edu.sa (D.O.); 2 Department of Physics, College of Science, King Faisal University, P.O. Box 400, Al-Ahsa 31982, Saudi Arabia; 3 Egyptian Petroleum Research Institute, Nasr City, P.O. Box 11727, Cairo 11765, Egypt; 4 Physics Department, Faculty of Science, Assiut University, P.O. Box 71515, Assiut 71516, Egypt

**Keywords:** optical active nanohybrids, photocatalytic degradation of dyes in sunlight, magnetic-nonmagnetic nanohybrids, nanolayers and nanocomposites

## Abstract

Energy and water related problems have attracted strong attention from scientists across the world because of deficient energy and water pollution. Following this line, new strategy depended on preparing nanolayers of Al/Zn and magnetic nanoparticles of cobalt iron oxides nanocomposite in addition to long chains of hydrocarbons of stearic acid to be used as roofs, fillers and pillars; respectively, to design optical-active nanohybrids in sunlight for removing the colored pollutants from water in few minutes. By using long chains of hydrocarbons of stearic acid, X-ray diffraction (XRD) results and TEM images showed expansion of the interlayered spacing from 0.76 nm to 2.02 nm and insertion of magnetic nanoparticles among the nanolayers of Al/Zn. The optical properties and activities showed that the nanohybrid structure based on zinc oxide led to clear reduction of the band gap energy from 3.3 eV to 2.75 eV to be effective in sunlight. Photocatalytic degradation of the dye of acid green 1 confirmed the high activity of the prepared zinc oxide nanohybrids because of a complete removal of the dye after ten minutes in sunlight. Finally, this strategy was effective for producing photo-active nanohybrids for using renewable and non-polluting energy for purifying water.

## 1. Introduction

Dyeing industry has annually used more than hundred thousand types of dyes. In addition, over 700,000 tons of dyes are produced worldwide [1]. In recent decades, the removal of synthetic dyes became international challenge because of the health problems in humans and animals due to these colorful effluents [2,3,4,5]. In addition, deficient energy is another challenge for the scientific society. These international challenges are produced through the rapidly growing population and industries which led to these energy and environment related problems. Many scientists have used energy to solve the problem of water pollution leading to increasing the problem of deficient energy. For saving the energy, the scientists tried to discover different techniques depending on purifying water by renewable and non-polluting energy. One of the most familiar non-polluting resources for energy is Sunlight. The solar energy can produce strong oxidizing agents for converting the industrial pollutants to carbon dioxide and water through exciting active photocatalysts. Most of the organic dyes such as textile dyes and surfactants are not easily biodegradable. Therefore, they belong to the colored hazardous pollutants. Photocatalytic degradation seems as one of the benign solutions for purifying water from organic dyes using photocatalysts and sunlight [6,7,8]. For solving these environmental problems, semiconductors photo-catalysts are very familiar in this trend [9,10,11,12]. 

Although, titanium oxide was one of the most famous photo-catalysts in this field, their applications were limited because it can mainly absorb UV-light which considers 4% of the solar energy [13,14,15]. Therefore, zinc oxide is suggested to be an alternative photocatalyst to titanium oxide because it has large excitation binding energy of 60 meV in addition to a band gap of 3.37 eV. According to the results of Dindar and Icli [16], zinc oxide was more effective than titanium oxide in sunlight for the degradation of phenol. Many researchers confirmed this conclusion through comparing between titanium oxide and zinc oxide semiconductors through the advanced oxidation of wastewater [17,18,19,20,21,22]. However, low performance of zinc oxide was observed for photocatalytic degradation in many studies [23,24,25] because of the high rate of recombination reactions for the excited electrons and holes of zinc oxide which happened within nanoseconds and the low amount energy absorbed during the photocatalytic processes. These disadvantages decrease the importance of photocatalytic degradation processes in the market.

Several techniques have used for modifying the structure of zinc oxide to solve its problems through narrowing its band gap energy to be active in sunlight. Formation of nanostructures [2], combination with carbon nanorods and nanotubes [26] and introducing surface defects were used to be good solutions for improving the activity of zinc oxides. In addition, for preventing the disadvantages of zinc oxide, doping processes with transition elements in addition to morphological changes [27] were studied to be suitable solutions for increasing the performance of zinc oxide for photocatalytic degradation of pollutants. In this trend, the optical properties and activity of zinc oxide were developed through the morphological changes from nanoparticles [28] to nanorods [29]. In addition, the zinc oxides nanotubes [30], and nanowires [31] were suggested to be active photocatalysts [32].

Many researchers have used transition elements for doping zinc oxide to become effective photocatalysts [33,34,35,36,37]. Insertion of sulfur inside the structure of ZnO im-proved the charges separation through preventing the recombination process between electrons and holes [38]. The results of Adeel et al. showed high photocatalytic degradation of rhodamine blue and methylene blue under UV irradiation using ZnO films which modified by the addition of Ag and Al [39]. Introduction of nitrogen using micro-emulsion method increased the optical properties and activity of ZnO nanospheres [40]. In addition, several studies concluded that the addition of aluminum and iron as dopants inside ZnO structures converted their transparent thin films to be useful for photocatalytic applications and solar cell [41,42,43,44,45,46]. This positive effect of addition of aluminum inside zinc oxide was confirmed by our previous research [12]. Thus, the current research aims to improve the photocatalytic activity of ZnO structure through building nanohybrids based on organic, magnetic and inorganic species by an unconventional technique. In the conventional methods [43], multi-steps were used for mixing one or two elements for zinc oxides. However, it is difficult to obtain a homogenous distribution for all dopants in the matrix of ZnO in this way.

The nanosize spinel ferrite particles CoFe_2_O_4_ recently have received considerable attention because of their remarkable photocatalytic properties. Although, a lot of research has been carried out for the photocatalytic performance of cobalt ferrite nanoparticles, there are no articles for using CoFe_2_O_4_ as filler for zinc oxide structure. In addition, because of the low band gap energy of cobalt iron oxide CoFe_2_O_4_ (1.32 eV), it considers excellent dopant and filler for reducing the band gap energy for zinc oxide.

Following this trend, the current study has used new strategy for building zinc oxide nanohybrids to be effective in sunlight for purifying water from pollutants. In this strategy, series of zinc oxides nanohybrids based on magnetic, inorganic and organic species were prepared through building inorganic-magnetic-organic and inorganic-magnetic nanohybrids. The inorganic-magnetic-organic nanohybrids were formed by intercalation reactions of long chains of stearic acids and inserting magnetic nanoparticles of cobalt iron oxides nanocomposites inside the nanolayered structures of zinc and aluminum. Organic species are used as pillars to widen the interlayered spacing of the nanolayered structures to allow for the magnetic nanoparticles for inserting between the nanolayers of zinc and aluminum. In addition, inorganic-magnetic nanohybrids were prepared without the long chains of organic acid to identify the role and the effect of organic species. These nanohybrids were used for producing zinc oxide nanohybrids by thermal treatment. Zinc oxide nanohybrids were tested for purifying water using sunlight through photocatalytic degradation of the colored pollutants. In the same time, the optical properties and activities of the nanohybrids were studied and compared with the conventional photocatalysts. This strategy is useful for doping photo-active materials with multi-dopants in special arrangements in the nano scale producing nanocomposites and nanohybrids with unusual and unique properties. 

## 2. Results

### 2.1. Characterization of the Prepared Filler

**Figure 1:**
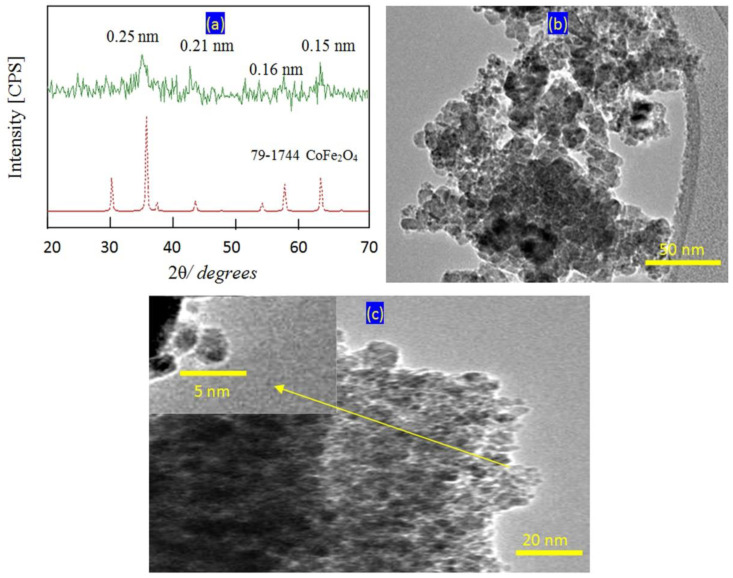
The prepared cobalt iron oxides nanocomposite (**a**) X-ray diffraction pattern, (**b**) TEM image at 50 nm and (**c**) TEM image at 20 nm (inset at 5 nm).

Very fine nanoparticles of cobalt iron oxides nanocomposite were prepared and characterized to be suitable for using as a filler and inserting among the nanolayers of the nanolayered structures. In this trend, X-ray diffraction has used for confirming the structure of the prepared cobalt iron oxides nanocomposite. Figure 1a showed X-ray diffraction pattern of the prepared cobalt iron oxides nanocomposite.

X-ray diffraction pattern showed weak peaks at 2Ѳ = 35.56°, 41.60°, 57.28° and 62.9° agreeing with d-spacings at 0.25 nm, 0.21 nm, 0.16 nm and 0.15 nm; respectively. By comparing with the standard diffraction pattern of JCPDS 79-1744, Figure 1a revealed that the prepared cobalt iron oxides have CoFe_2_O_4_ structure. Transmission electron microscopy has used for measuring the nano size of the particles of the prepared cobalt iron oxides. Figure 1b showed strong aggregates of nanoparticles because of the magnetic behavior of the cobalt iron oxides. By magnification, very fine nanoparticles were observed in Figure 1c. The more magnification showed that the size of the particles of the prepared cobalt iron oxides is less than 5 nm as seen in Figure 1c (inset).

### 2.2. Design of Organic-Inorganic-Magnetic Nanohybrids

**Figure 2:**
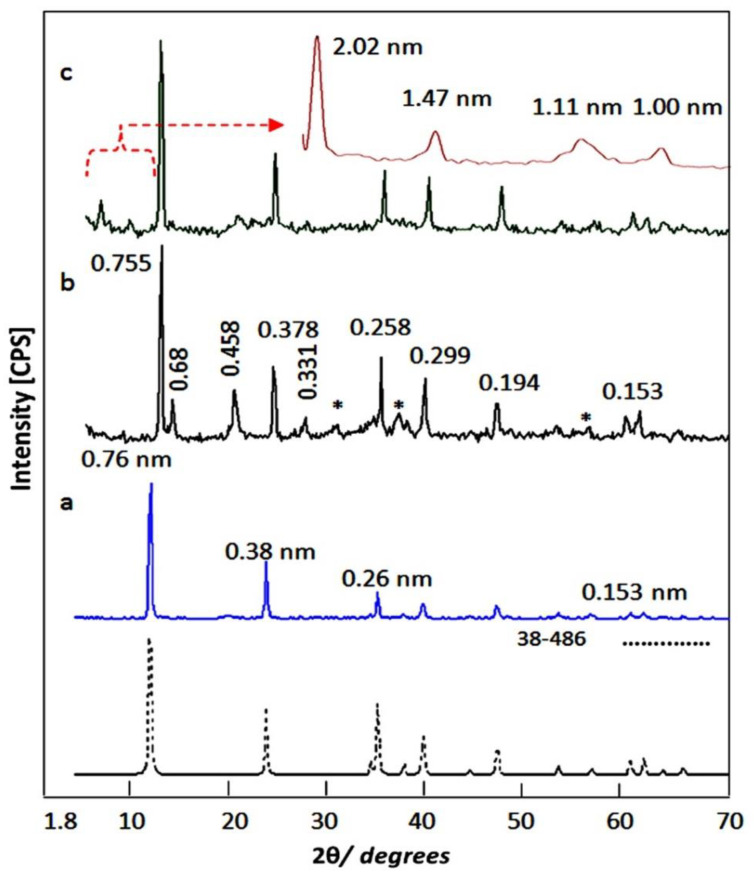
X-ray diffraction patterns of: (**a**) the pure Al/Zn nanolayered structure, (**b**) the nanohybrid ZNH-1 (* is due to cobalt iron oxides) and (**c**) the nanohybrid ZNH-2.

Organic-inorganic-magnetic nanohybrids appear to be very creative because they can produce unlimited set of known or unknown properties. In this way, nanohybrids were designed by combination between zero dimensional nanoparticles of magnetic nanocomposite and two dimensional nanolayered structures in addition to long chains of organic acid. This combination was achieved in an order arrangement through building Al/Zn nanolayered structures which have cationic nanolayers. In presence of stearic acid (CH_3_(CH_2_)_16_COO^−^), the long chains of the aliphatic acid were intercalated among the nanolayers for neutralizing their positive charges. At the same time, the long chains of stearate anions were working as pillars for building the nanolayered structures. In addition, these pillars expanded and widened the interlayered spacing among the nanolayers to produce enough space for existing magnetic nanoparticles of cobalt iron oxides nanocomposite. To indicate the positive role of organic species for designing this nanohybrid, pure Al/Zn nanolayered structure was prepared for comparison. In addition, Al/Zn nanolayered structure was modified by the nanoparticles of cobalt iron oxides nanocomposites without organic species to study the inorganic-magnetic nanohybrid. X-ray diffraction patterns of the prepared nanolayered structures and nanohybrids were displayed in Figure 2.

Figure 2a showed the X-ray diffraction pattern of the pure Al/Zn nanolayered structure AlZO. Sharp and symmetric peaks were observed at 2Ѳ = 11.62°, 23.36°, and 34.54° aligning with d-spacing of 0.76 nm, 0.38 nm and 0.26 nm. These peaks are due to the reflections of the main planes [003], [006] and [009]. The clear arrangement between these reflections (0.76 nm = 2 × 0.38 nm = 3 × 0.26 nm) confirmed formation of the nanolayered structures of the natural hydrotalcite (JCPDS file No. 37-629) and zinc aluminum carbonate hydroxide hydrate (JCPDS file No. 38-486). The other reflections of the planes [012], [015], [110] and [113] of the nanolayered structures of the natural hydrotalcite were observed at 2 theta 39.16°, 46.56°, 60.05° and 61.44° and matching with d-spacing 0.23 nm, 0.19 nm, 0.17 nm, 153 and 0.150 nm. The crystal parameters (a, c) could be calculated depending on the d-spacing of the planes [003] and [110]; respectively. The first parameter was 2 × d_[110]_ = 0.306 nm. It means that the average distance between Zn cation and Al cation is 0.306 nm agreeing with the previous published data of zinc aluminum carbonate hydroxide hydrate (JCPDS file No. 38-486). The second parameter was assessed by 3 × d_[003]_ = 2.28 nm. It was similar to that reported for the natural hydrotalcite.

With incorporating the nanoparticles of cobalt iron oxides nanocomposite with the pure Al/Zn nanolayered structures without organic species, ZNH-1 was formed to build the inorganic-magnetic nanohybrid. Figure 2b showed a little shift for the main peaks of the nanolayered structures in addition to appearing new peaks after building the nanohybrid ZNH-1.

The crystal parameter (a) which depends on the reflection of the plane [110] did not change. In the same time, a little change was observed for the parameter (c) from 2.280 nm to 2.265 nm. It means that the nanohybrid ZNH-1 has the same nanolayered structure with new phases. The appearance of clear peaks at 2 theta 13.01°, 19.48° and 26.78° with d-spacing 0.68 nm, 0.455 nm and 0.331 nm indicated growth of new phase hydrozincite Zn_5_(OH)_6_(CO_3_)_2_. In addition, the presence of magnetic nanoparticles was confirmed by observing the characteristic peaks of cobalt iron oxides which marked with (*) in Figure 2b. It means that the magnetic nanoparticles were supported on the external surface of the nanolayered structure because of their size. The small interlayered spacing between the nanolayers is unsuitable for inserting the nanoparticles because of their size. In addition, XRD results showed their peaks indicating that these nanoparticles are not covered or coated by the nanolayers.

**Figure 3:**
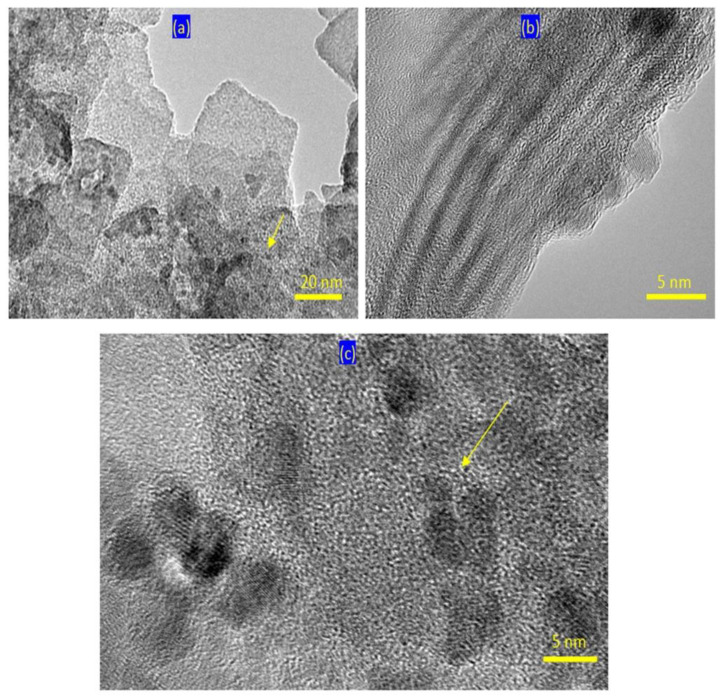
TEM images of the nanohybrid ZNH-2: (**a**) the first location at 20 nm, (**b**) the second location at 5 nm and (**c**) the first location at 5 nm (Arrows due to very fine nanoparticles).

By intercalating the long chains of stearic acid (CH_3_(CH_2_)_16_COO^−^) with the Al/Zn nanolayered structures in presence of the nanoparticles of cobalt iron oxides nanocomposite, the organic-inorganic-magnetic nanohybrid ZNH-2 was formed through host-guest interaction. X-ray diffraction pattern of ZNH-2, which was displayed in Figure 2c, showed new peaks at low 2Ѳ in addition to the original peaks of the nanolayered structures. With noting that the peaks of the nanoparticles of magnetic nanocomposite became unclear. The new peaks of the nanohybrid ZNH-2 became clearer after measuring the X-ray diffraction at low range of 2Ѳ from 4–10 as seen in Figure 2c (inset). Sharp peak was observed at 2.02 nm indicating that the interlayered spacing of the nanolayered structure expanded and widened from 0.755 nm to 2.02 nm. This spacing could allow for the nanoparticles of cobalt iron oxides to intercalate among the nanolayers of the nanolayered structure because the peaks of cobalt iron oxides are not clear in Figure 2c. It means that the nanohybrid ZNH-2 consists of nanolayered structures having organic species and magnetic nanoparticles. This finding was confirmed by transmission electron microscopy (TEM). TEM images of the nanohybrid ZNH-2 were displayed in Figure 3. Figure 3a showed that the nanohybrid ZNH-2 has nano-platelets with size less than 50 nm. In addition, very small black dotes, which marked by arrow, were observed in Figure 3a representing the magnetic nanoparticles of cobalt iron oxides nanocomposite. By magnification, Figure 3c confirmed the presence of the magnetic nanoparticles. In addition, Figure 3b confirmed the nanolayered structures with interlayered spacing 2 nm.

**Figure 4:**
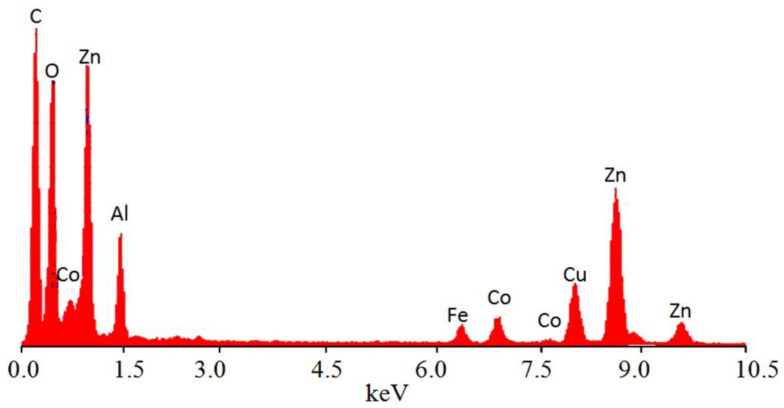
EDX spectrum of the nanohybrid ZNH-2.

The presence of magnetic, inorganic elements and organic species was confirmed by energy dispersive X-ray spectrometry (EDX) analysis. Although EDX spectra indicate the local data of the different elements in the outermost layers of the platelets of the nanohybrid, the magnetic elements were identified by clear peaks for cobalt and iron as seen in Figure 4. In addition, the inorganic elements (zinc, aluminum and oxygen) were observed by sharp peaks in Figure 4. In addition, a strong peak for carbon was observed confirming the presence of organic species.

**Figure 5:**
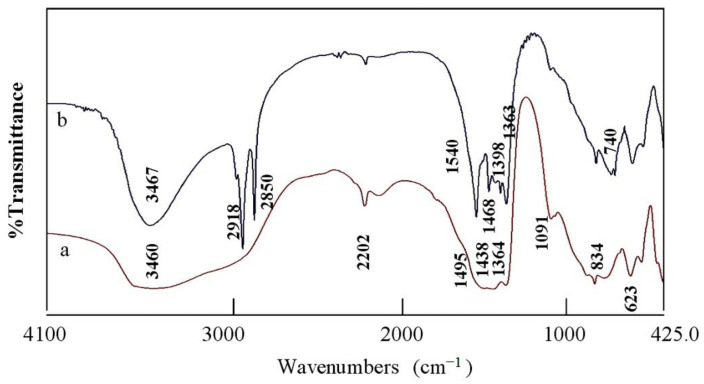
FT-IR spectra of: (**a**) the nanohybrid ZNH-1 and (**b**) the nanohybrid ZNH-2.

The Fourier Transform infrared (FT-IR) spectroscopy was applied to compare between the function groups of the nanohybrids ZNH-1 and NH-2 as shown in Figure 5. For the nanohybrid ZNH-1, Figure 5a showed absorption band at 3460 cm^−1^ indicating the stretching mode of hydroxyl groups [11,47]. The large broadness of the OH band between 3500 cm^−1^ and 3400 cm^−1^ indicated the presence of two types of hydroxyl groups which belonged to the nanolayered structures and hydrozincite phase [48]. The shoulder which recorded around 3000 cm^−1^ is due to the hydrogen bonds of carbonate anions [49,50]. The bands at 1495 cm^−1^ and 1364 cm^−1^ should be due to vibrational mode of the interlayer carbonate anions [51]. The other band at 1438 cm^−1^ represented the carbonate anions of hydrozincite phase agreeing with XRD results [52]. The weak band at 2202 cm^−1^ is due to presence of cyanate anions indicating that the nanohybrid ZNH-1 has two types of interlayered anions [53]. The bands observed below 1000 cm^−1^ could be ascribed to Zn-O and Al-O [54,55].

For the nanohybrid ZNH-2, Figure 5b confirmed the presence of long chains of organic species in the IR spectrum because the stretch absorption of carbon–hydrogen was observed by sharp peaks at 2918 cm^−1^ and 2850 cm^−1^ [56]. In addition, the bending mode of the carbon–hydrogen was clear through observing band at 1468 cm^−1^. The symmetric stretching vibration of carboxylate, which belonged to the aliphatic acid, was observed at 1540 cm^−1^ [56]. Furthermore, the absorption at 1398 cm^−1^ is assigned to the asymmetric stretching vibration of carboxylate. In addition, the absorption band of the hydroxyl groups of the nanolayered structure was observed at 3467 cm^−1^ [56]. In the same trend, the presence of long chains of organic species inside the nanohybrid ZNH-2 was confirmed by thermal analyses.

**Figure 6:**
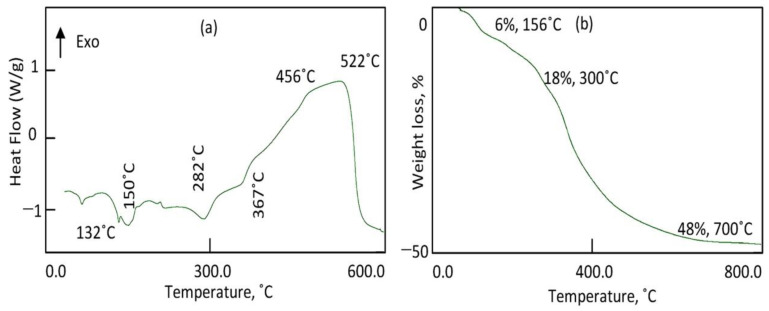
Thermal analyses of the nanohybrid ZNH-2: (**a**) differential scanning calorimetric and (**b**) thermal gravimetric analysis.

The thermal gravimetric analysis and differential scanning calorimetric (TGA-DSC) curves showed that the degradation occurs through a continuous process with various mass rate losses, depending upon the nature of the interlayer species. The DSC curve of the nanohybrid ZNH-2 showed two series of peaks as shown in Figure 6a. The first series is endothermic peaks at 132 °C, 150 °C and 282 °C which are ascribed to the removal of water and inorganic anions. The second series is exothermic peaks at 367 °C, 456 °C and 522 °C representing the oxidation reactions of the organic species. From the TG curve, Figure 6b showed that the nanohybrid ZNH-2 has 48% of unstable components. The weight loss 18%, which happened up to 300 °C, represents the internal content of water and inorganic anions inside the nanohybrid ZNH-2. In the same way, the weight loss 30%, which occurred up to 700 °C is due to the internal content of organic species inside the nanohybrid ZNH-2. It means that ZNH-2 is mainly inorganic-magnetic-organic nanohybrid.

### 2.3. Design of Nanohybrids Based on Oxides

The main reason for designing nanohybrids with organic and inorganic species is directed to produce stable and effective zinc oxides nanohybrids and nanocomposites with distinguished properties. Therefore, the prepared nanohybrids were thermally treated at 500 °C to remove unstable species and create new active sites.

**Figure 7:**
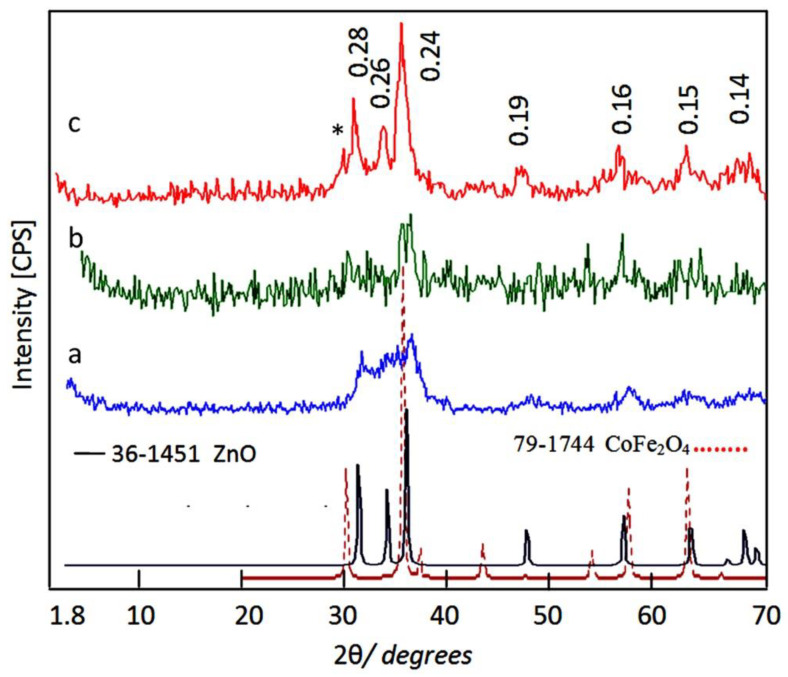
X-ray diffraction patterns of: (**a**) AlZO-500, (**b**) ZNH-1-500 and (**c**) ZNH-2-500 (* is due to cobalt iron oxides).

X-ray diffraction has used to identify the produced structures from the calcination of the nanohybrids. Figure 7 showed X-ray diffraction patterns of AlZO-500, ZNH-1-500 and ZNH-2-500. The XRD pattern of AlZO-500 exhibited new weak peaks at 2Ѳ = 32.01°, 34.32°, 36.49°, 47.71°, 7.05°, and 62.81° in addition to the original peaks of the nanolayered structures disappeared as shown in Figure 7a. By comparing with the diffraction lines of the zinc oxide crystal (JCPDS No. 36-1451) and the standard entire diffraction pattern of zincite phase (JCPDS No. 75-576), AlZO-500 has similar structure for zinc oxide. However, the broad and diffuse peaks of AlZO-500 indicated that the structure of AlZO-500 is not pure because of the presence of the amorphous structure of aluminum oxide inside the zincite phase. In case of the nanohybrid ZNH-1-500, X-ray diffraction patterns showed that this structure tends to be amorphous because the peaks of zinc oxide became unclear. Only two peaks of the zinc oxide, which observed at 2 theta 36.30 and 62.92, were identified in the X-ray diffraction pattern of ZNH-1-500. In addition, the weak peaks of cobalt iron oxides were also observed at 2Ѳ = 30.29°, 35.65°, 37.78°, 40.36°, 53.77°, 57.28° and 64.86° in Figure 7b. It means that ZNH-1-500 has two different types of structures; cobalt iron oxides and the Al-doped zinc oxides. For the nanohybrid ZNH-2-500, Figure 7c showed clear and sharp peaks at 0.28 nm, 0.26 nm and 0.24 nm indicating crystalline structure. In addition, weak peaks were observed at 0.19 nm, 0.16 nm, 0.15 nm and 0.14 nm. These diffraction lines agree with the peaks of the zinc oxide crystal (JCPDS No. 36-1451) and the standard entire diffraction pattern of zincite phase (JCPDS No. 75-576). In addition, weak peak was observed at 0.30 nm and marked with (*) in Figure 7c. In the same time, Figure 7c revealed that the characteristic peak of cobalt iron oxides at 0.25 nm overlapped with the peak of zinc oxide at 0.26 nm. These XRD results can conclude that ZNH-2-500 has zincite phase doping with aluminum and cobalt iron oxides.

**Figure 8:**
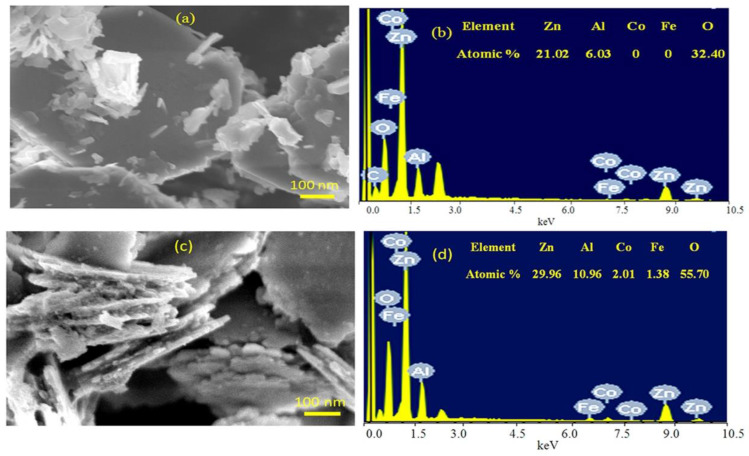
ZNH-1-500: (**a**) SEM images, (**b**) EDX spectrum and ZNH-2-500: (**c**) SEM images, (**d**) EDX spectrum.

These results of X-ray diffraction were confirmed by scanning electron microscopy. Figure 8 showed SEM images and EDX spectra of the nanohybrids ZNH-1-500 and ZNH-2-500.

Figure 8a showed that ZNH-1-500 has two structures; white nanoparticles and large black plates agreeing with XRD results. The chemical composition of the black plates was determined by EDX analysis. Figure 8b showed that the black plates are zinc oxide doped with aluminum with Zn/Al molar ratio = 3.5 agreeing with the nanolayered structure of Al/Zn LDH. It means that the other white nanoparticles are the cobalt iron oxide nanocomposite.

Figure 8c showed one phase for ZNH-2-500 as seen in SEM image. The chemical composition of ZNH-2-500, which determined by EXD analysis, confirmed formation of zinc oxide doped with Al, Co and Fe agreeing with XRD results. Figure 8d indicated that 2.01% of Co and 1.38% of Fe inserted in the ZnO structure in addition to presence of 10.96% of Al.

**Figure 9:**
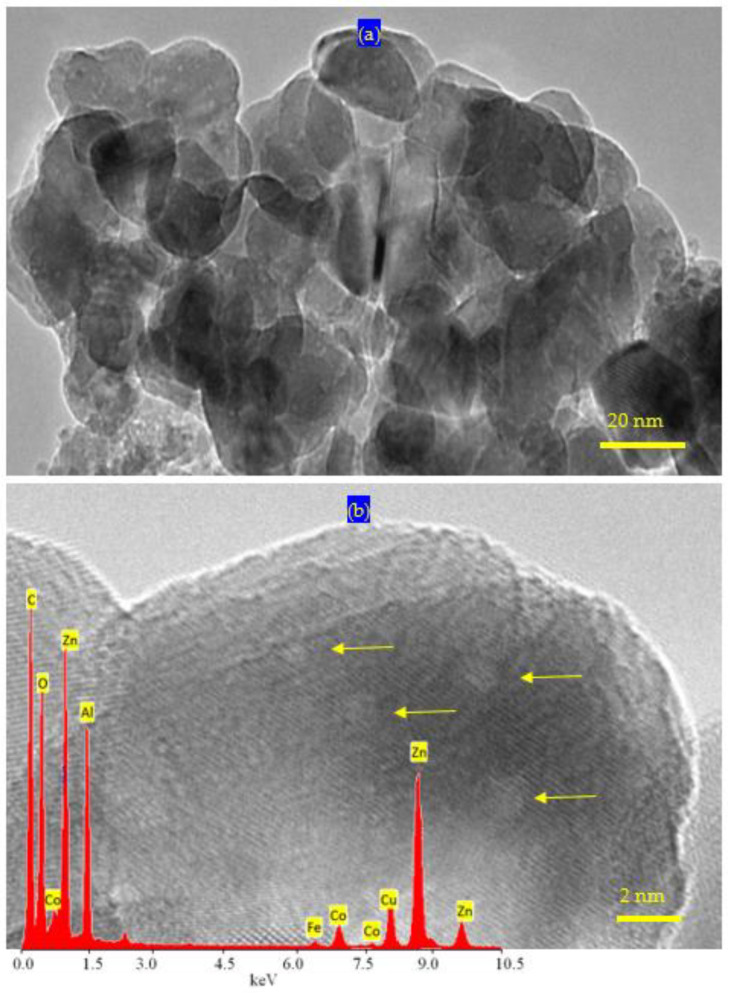
TEM images of ZNH-2-500: (**a**) 20 nm and (**b**) 2 nm (inset: EDX spectrum); the arrows are due to very fine nanoparticles.

TEM images of ZNH-2-500 confirmed this finding as shown in Figure 9. Clear nanoparticles were observed for ZNH-2-500 as seen in Figure 9a. It indicated that the width of ZNH-2-500 is 20 nm. In addition, very fine white spots were observed and marked by arrow on the surface of the nanoparticles. These spots represent the cobalt iron oxides nanocomposites involved inside ZnO structure. These white spots became clearer by magnification as seen in Figure 9b. Figure 9b confirmed the presence of white indicating that the size of cobalt iron oxides is less than 2 nm. Energy dispersive X-ray spectrometry (EDX) analysis of ZNH-2-500 confirmed the presence of magnetic, elements through observing two weak peaks for cobalt and iron as seen in Figure 9b (inset). In addition, the inorganic elements (zinc, aluminum and oxygen) were observed by sharp peaks in Figure 9b (inset).

**Figure 10:**
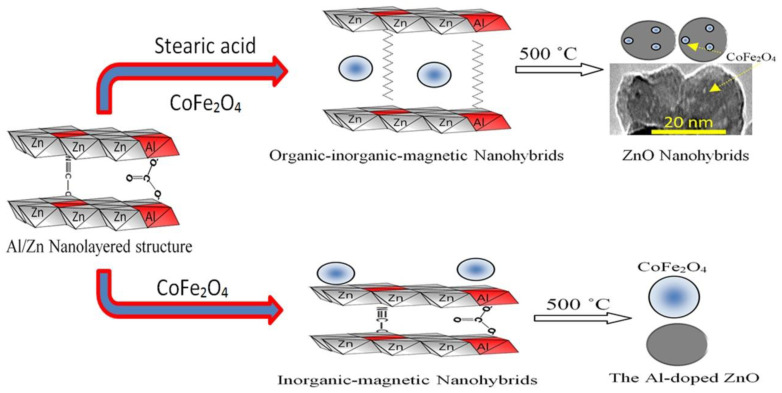
Schematic representation of zinc oxide nanohybrids based on organic-inorganic-magnetic and inorganic-magnetic nanohybrids.

According to the results of XRD and the images of SEM and TEM, the nanohybrids based on zinc oxide was clear from the thermal decomposition of the organic-inorganic-magnetic nanohybrid as shown in Figure 10.

Figure 10 showed schematic representation for transforming the organic-inorganic-magnetic nanohybrid to zinc oxide nanohybrids. In addition, Figure 10 indicated the difference between the thermal decomposition of the organic-inorganic-magnetic nanohybrid and the inorganic-magnetic nanohybrid. The presence of magnetic nanoparticles of cobalt iron oxides nanocomposite inside the nanolayered structure of Al/Zn gave chance for incorporation of cobalt iron oxides nanoparticles with the produced nanoparticles of the Al-doped ZnO during the thermal decomposition of organic species and the crystallization process of zinc oxide creating new optical active sites ZNH-2-500. In case of the inorganic-magnetic nanohybrid, the presence of the magnetic nanoparticles of cobalt iron oxides on the external surface of the nanolayered structure did not allow for the incorporation process with the produced Al-doped ZnO. Therefore, XRD results and SEM images of ZNH-1-500 showed two crystalline structures for zinc oxide and cobalt iron oxides.

### 2.4. Optical Properties

Zinc oxide is familiar for the researchers in the field of optical application as one of the most famous photo-active materials. However, its optical applications are concentrated in the UV-region. Therefore, many studies were published in literature for developing the structure and the morphology of zinc oxide to advance its optical behavior through increasing the range of its absorbance and decreasing its band gap energy.

In this way, the optical absorbance and the band gap energy of the prepared nanocomposites were studied and compared by using the UV-Vis absorption technique which considered a powerful tool for providing important details about their optical properties.

**Figure 11:**
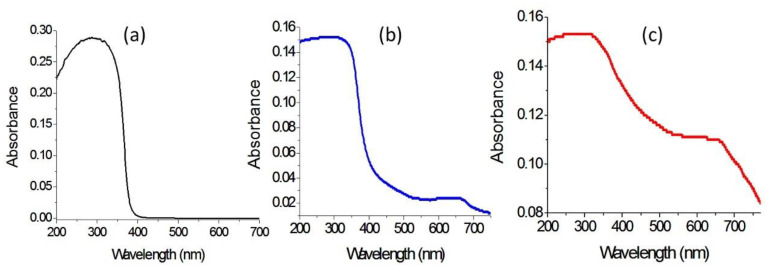
UV-Vis absorbance of (**a**) AlZO-500, (**b**) ZNH-1-500 and (**c**) ZNH-2-500.

Figure 11 showed the UV-Vis absorbance of AlZO-500, ZNH-1-500 and ZNH-2-500. Figure 11a indicated that AlZO-500 is active in the UV region because it has absorption in the range of wavelength 200–350 nm. At the same time, there is no absorption in the visible region above 400 nm. By modifying the structure of AlZO-500 through combining with magnetic nanocomposites, the optical properties of ZNH-1-500 improved as shown in Figure 11b. New absorbance band was observed in the visible region at 650 nm. The weakness of the new peak means that the introduction of magnetic nanocomposites with the Al-doped zinc oxide started to improve its optical properties. This positive effect of the magnetic nanocomposite increased through building the organic-inorganic-magnetic nanohybrid structures because Figure 11c showed clear absorbance for ZNH-2-500 starting from 750 nm to 200 nm with two maxima at 650 nm and 350 nm. It means that the intercalation of magnetic nanoparticles inside the interlayered space of the nanohybrid led to good and order dispersion inside the structure of zinc oxide after calcination creating new optical active centers for ZnO.

**Figure 12:**
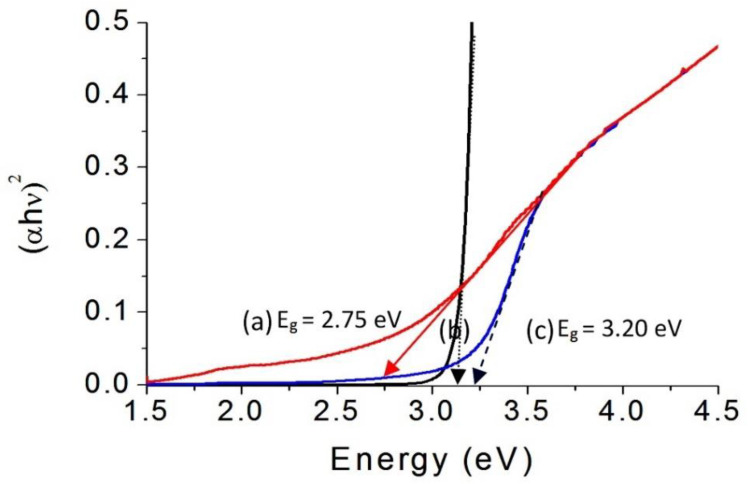
Band gap of (**a**) ZNH-2-500, (**b**) ZNH-1-500 and (**c**) AlZO-500.

This finding was confirmed by calculating their band gap energy. The band gap energy was determined through plotting the relation between (αhν)^2^ and energy (hν) as shown in Figure 12. The band gap energy E_g_ of AlZO-500 was calculated by extending the straight line to the (hν) axis to obtain the optical band gap energy at (αhν)^2^ of 0. It showed 3.20 eV as seen in Figure 12c indicating a little shift from the band gap of pure ZnO because of the doping of aluminum inside the zinc oxide structure. In case of ZNH-1-500, a little change was observed for the band gap energy because Figure 12b showed changing from 3.20 eV to 3.15 eV. The weakness effect of the magnetic nanocomposite for the absorbance of ZNH-1-500 did not strongly affect the band gap energy. Meanwhile, the strong effect of the magnetic nanocomposite on the absorbance of ZNH-2-500 was clear for narrowing the band gap energy to be 2.75 eV as shown in Figure 12a. By comparing with the pure zinc oxide, the narrowing was clearer because the reduction was from 3.30 eV to 2.75 eV indicating that the organic-inorganic -magnetic nanohybrids has a strong positive effect on the optical properties of zinc oxide.

### 2.5. Optical Activity

**Figure 13:**
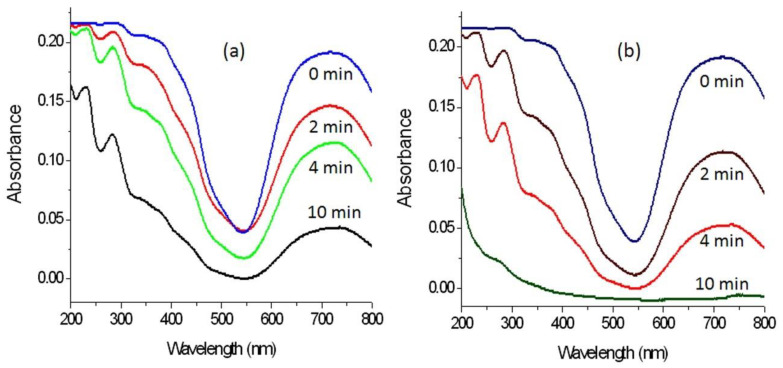
Photocatalytic degradation of Acid green 1 at different times of sunlight exposure in presence of: (**a**) ZNH-1-500 and (**b**) ZNH-2-500.

It is known that the improvement of the optical properties of the products of zinc oxides leads to positive effects for their photo activities. In order to indicate these positive effects, the prepared products have used as photocatalysts to be appropriate means for increasing the photocatalytic activity of zinc oxide to decompose and remove pollutants by sunlight in short time. In this way, the green dye of Acid green 1 was used as an example for colored pollutants. The photo activities of zinc oxides (doped or un-doped), and their products based on the nanohybrids structure were studied through photocatalytic degradation of Acid green 1. By irradiating the aqueous solution of Acid green 1 with the sunlight in the presence of the photocatalyst and measuring the absorbance of the liquid portion after the irradiation for certain minutes, the reduction in the absorption of the green dye at wavelength 714 nm indicated the degradation of the main structure of the pollutant, while the degradation of the naphthyl rings in the dye could be followed from the absorption peaks at 322 nm, 280 nm, and 230 nm as shown in Figure 13a,b.

Blank experiment, which was performed without photocatalyst, indicated high stability of the Acid green 1 toward the light irradiation. The photocatalytic degradation of the green dye was investigated as a function of the sunlight irradiation time in the presence of the photocatalyst as seen in Figure 13. Figure 13a showed the photocatalytic degradation of Acid green 1 under sunlight in the presence of ZNH-1-500. By increasing the irradiation time, the photocatalytic degradation of Acid green 1 increased. 75% of removal of the green color was observed after 10 min of sunlight irradiation time for ZNH-1-500. In case of using ZNH-2-500, the activity became higher as shown in Figure 13b. A complete photocatalytic degradation of the Acid green 1 was achieved after 10 min of sunlight irradiation time. It means that ZNH-2-500 is active in sunlight because it completely destroyed the green dye at shorter time.

The high performance of the zinc oxide nanohybrids ZNH-1-500 and ZNH-2-500 was clear after comparison with the AlZO-500 and the pure zinc oxide. Where, the complete removal of the green dye was happened after 360 min of solar energy in presence of AlZO-500. In case of the pure zinc oxide, the complete removal of the green dyes was achieved after 840 min of sunlight irradiation time. It means that the zinc oxide nanohybrids became very active in sunlight.

In order to indicate the effect of the organic species on the optical activity, the kinetics of photocatalytic decolorization and degradation of Acid green 1 were studied for both ZNH-1-500 and ZNH-2-500 by the following equation:

ln([C_o_]/[C]) = kt (1)

The rate reaction constant is k. The initial concentration of Acid green 1, which was expressed by the absorbance at time equal zero, is coded as [C_o_]. The concentration of acid green 1 at different times is coded as [C]. By plotting the irradiation time in minutes against ln([C_o_]/[C]), the diagrams could be employed for kinetically determining the type of reactions.

**Figure 14:**
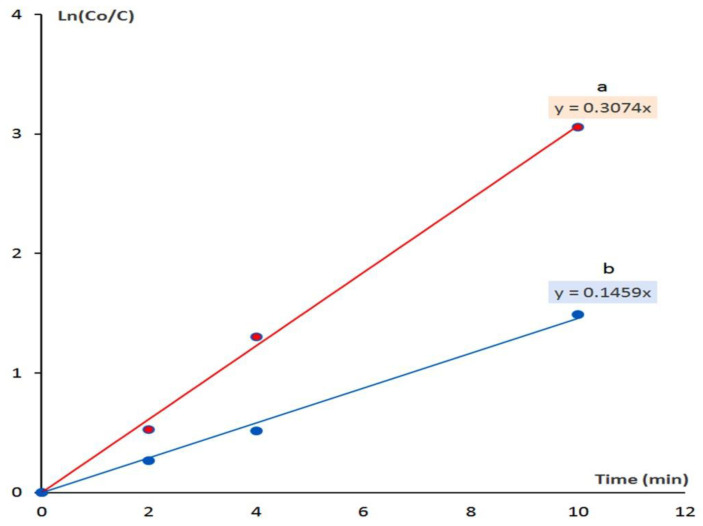
Kinetics study of the Photocatalytic degradation of Acid green 1 in presence of: (**a**) ZNH-2-500 and (**b**) ZNH-1-500.

According to Figure 14, the diagrams indicated that the photocatalytic degradation and decolorization of Acid green 1 are pseudo-first-order reactions in case of using both ZNH-1-500 and ZNH-2-500.

Figure 14a showed that the rate reaction constant of the photocatalytic degradation of Acid green 1 in presence of ZNH-2-500 is 0.307 min^−1^. By using ZNH-1-500, the reaction became slower because the rate reaction constant increased to be 0.146 min^−1^ as shown in Figure 14b. The kinetics study concluded that the rate of photocatalytic degradation of Acid green 1 in presence of ZNH-2-500 increased to become two times higher than that of ZNH-1-500. This relation showed the important role of organic species for producing the high performance of ZNH-2-500. It means that the zinc oxide nanohybrid, which based on inorganic-magnetic-organic nanohybrid ZNH-2-500, is better than the zinc oxide nanohybrid which produced from inorganic-magnetic nanohybrid. In addition, the same results were observed for ZNH-2-500 after repeating the experiments two times with the fresh sample of the green dye indicating high recyclability of the produced photocatalyst.

## 3. Discussion

The fast photocatalytic degradation of the green dyes in sunlight showed the excellent activity of the prepared zinc oxide nanohybrid ZNH-2-500 which produced from inorganic-magnetic-organic nanohybrids. The high performance of ZNH-2-500 can be explained through the novel strategy for building the nanohybrid structure of ZNH-2-500. The intercalation of the fine nanoparticles of CoFe_2_O_4_ nanocomposite among the nanolayers of Al/Zn gave good chance for incorporation of this nanocomposite with zinc oxide structures during the crystallization process. Therefore, ZNH-2-500 has well-crystalline structure for zinc oxide and there are no peaks for aluminum and cobalt iron oxides. This good incorporation of CoFe_2_O_4_ nanocomposite with the crystals of zinc oxide is failed for the sample ZNH-1-500 because the nanoparticles of CoFe_2_O_4_ nanocomposite could not intercalate among the nanolayers of Al/Zn, but supported on the external surface of the plates of Al/Zn. The good incorporation of CoFe_2_O_4_ nanocomposite with the crystals of zinc oxide which doped with aluminum created new optical active centers inside zinc oxide nanohybrid ZNH-2-500 and caused reduction for its band gap energy to be very effective in sunlight because of the low band gap energy of CoFe_2_O_4_ (1.32 eV) [57]. In the same time, some sites of Zn in zinc oxide are occupied by CoFe_2_O_4_ atoms producing new optical active centers called shallow traps between the valance band and conduction band leading to decreasing for the band gap energy [3,58].

This low band gap energy and the small size of the nanoparticles of the zinc oxide nanohybrid ZNH-2-500 have strong effect on the mechanism of the photocatalytic degradation process of the green dyes. The mechanism of the photocatalytic degradation process in sunlight depends on two important stages [59,60,61].

The first stage is production of strong oxidizing agent as follow:

- By sunlight, the surface of Photocatalyst was excited through absorption of enough energy for transferring electrons from valance band to conduction band creating holes in the valance band.
  Sunlight + ZNH → h^+^ (valance band) + e^−^ (conduction band) (2)
- At the same time, oxygen molecules physically adsorbed on the surface of the photocatalyst ZNH which captured the electron from the conduction band producing ions.
  (O_2_)_ads_ + e^−^ → O_2_^•−^ (3)
- The holes in valance band neutralized the hydroxyl groups which produced from water molecules producing hydroxyl radicals.
  (H_2_O ↔ H^+^ + OH^−^) + h^+^ → OH^•^ + H^+^ (4)
- Neutralization of O_2_^•−^
  O_2_^−^ + H^+^ → HO_2_^•^ (5)
- Production of hydrogen peroxide
  2HO_2_^•^ → O_2_ + H_2_O_2_ (6)
- Hydrolysis of hydrogen peroxide
  H_2_O_2_ → OH^−^ + OH^•^ (7)
  The free radicals of hydroxyl groups (^•^OH) and superoxide radical anion (^•^O_2_^−^) are strong oxidizing agents. The second stage focuses on oxidation reactions of the pollutants by different kinds of the oxidizing agents.
- The superoxide radical anions oxidized and decomposed the green dyes
  AG1 + ^•^O2^−^ → Degradation compounds + CO_2_ + H_2_O (8)
- In addition, the holes started the degradation process of the pollutants
  AG1 + h^+^ → AG1^+^ → Degradation products (9)

The low band gap energy and the small size of the nanoparticles of ZNH-2-500 accelerated the first stage producing a large amount of oxidizing agents leading to fast removal of the green dyes. In addition, the band gap of ZNH-2-500 is not very small to accelerate the recombination reactions in addition to the shallow traps which help for separating between electrons and holes. Therefore, the degradation reaction continues as shown in Equations (8) and (9). By this way, the colored pollutants disappeared after ten minutes of sunlight exposure.

## 4. Materials and Methods

The hetero-structured hybrids such as inorganic–magnetic and organic–inorganic-magnetic systems are good candidates for creating unusual optical properties for zinc oxide which cannot be achieved by conventional methods.

**Figure 15:**
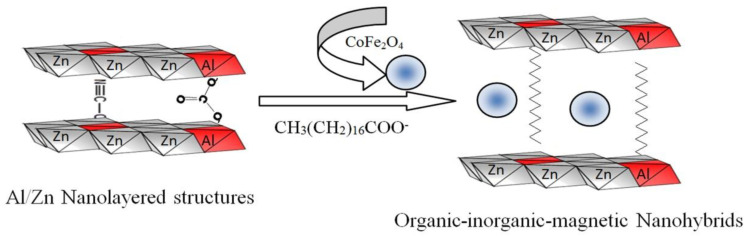
Schematic representation for building organic-inorganic-magnetic nanohybrids.

In order to build organic-inorganic-magnetic nanohybrids based on zinc oxide, three types of nanomaterials were prepared. The first type was very fine nanoparticles of cobalt iron oxides nanocomposite which were used as filler for the nanohybrids. The second one was nanolayered structures based on Al/Zn LDHs. The third type depended on the long chains of organic fatty acid to expand and widen the interlayered spacing of the nanolayered structures as shown in Figure 15. This widening and expansion can facilitate the insertion of magnetic nanoparticles among the nanolayers of Al/Zn LDHs. To study the important role of organic fatty acid, inorganic–magnetic nanohybrid was prepared without organic species.

### 4.1. Preparation of Magnetic Nanocomposites

A solvent thermal technique has used for preparing very fine nanoparticles of cobalt iron nanocomposite. Cobalt (II) acetate (0.096 M) and Iron (II) acetate (0.096 M) were reacted with 350 mL of methanol at room temperature for 5 h to produce Sol of methoxides as shown in the following equations:

CH_3_OH + Co(OCOCH_3_)_2_ → 2CH_3_COOH + Co(OCH_3_)_2_ (10)

CH_3_OH + Fe(OCOCH_3_)_2_ → 2CH_3_COOH + Fe(OCH_3_)_2_ (11)

Similar amount of ethanol was added for the mixture. To complete the reaction under super critical conditions of pressure and temperature, the mixture was placed inside autoclave. The mixture was heated by slow rate 1 °C/min. to reach 260 °C under high pressure 75 bar. The Sol converted to Gel by a poly-condensation or poly-esterification reactions that result in a dramatic increase in the viscosity of the solution during heating inside Autoclave. Poly-condensation reactions continue until the gel transformed into a solid mass of CoFe_2_O_4_

C_2_H_5_OH + CH_3_COOH → CH_3_COOC_2_H_5_ + H_2_O (12)

Co(OCH_3_)_2_ + 2H_2_O → —Co—OH + 2CH_3_OH (13)

Fe(OCH_3_)_2_ + 2H_2_O → —Fe—OH + 2CH_3_OH (14)

—Co—OH + HO—Fe— → —Co—O—Fe— + H_2_O (15)

At the final stage, the pressure was slowly released under flow of nitrogen to avoid oxidation reactions. Solvents were removed. In the same time, the temperature of the autoclave decreased to the room temperature. The fine powder of the product was easily collected.

### 4.2. Preparation of Nanohybrids and Nanolayered Structures

Three samples were prepared for designing nanolayered structure, inorganic-magnetic nanohybrid and organic-inorganic-magnetic nanohybrid. Nanolayered structure of Al/Zn LDH was prepared through mixing aqueous solutions (0.069 M) of aluminum nitrate with zinc nitrate in presence of 0.5 M of urea. The molar ratio of aluminum to zinc was 1:3. By keeping the temperature of the mixture at 80 °C, the nanolayers of LDH were precipitated during the hydrolysis of urea because the nature of reaction medium was gradually changed from acidic to alkaline. White precipitate was obtained after 12 h of reaction. It was filtrated and washed by distilled water. By drying at room temperature, the product was collected and coded by AlZO.

The inorganic- magnetic nanohybrid was prepared by the same procedure. With noting that the nanolayers of the Al/Zn LDH were precipitated in presence of 0.5 g of the prepared nanoparticles of cobalt iron oxides nanocomposite. The product was collected and codes by ZNH-1.

The organic-inorganic-magnetic nanohybrid was synthesized by adding 100 mL of the aqueous solution of 5% stearic acid sodium salt during building the Al/Zn nanolayered structure. In addition, 0.5 g of the prepared nanoparticles of cobalt iron oxides nanocomposite was mixed with the aqueous solution (0.069 M) of aluminum nitrate with zinc nitrate in presence of 0.5 M of urea. By keeping the temperature of the mixture at 80 °C, the product was obtained after 12 h of the reaction. After filtration and washing, the product was dried under vacuum at room temperature. The sample was coded by ZNH-2.

### 4.3. Preparation of Multi-Oxides Nanohybrids

The nanolayered structure of Al/Zn LDH was thermally treated at 500 °C to produce nanocomposite of zinc and aluminum oxides. It was represented by AlZO-500. By calcination at 500 °C, the nanohybrid ZNH-1 was converted to be stable nanohybrid composing of magnetic and non-magnetic oxides. It was represented by ZNH-1-500. The organic-inorganic-magnetic nanohybrid ZNH-2 was transformed to new structure of nanohybrid through the thermal treatment at 500 °C. It was represented by ZNH-2-500.

### 4.4. Physical Characterization

Nanolayered structures and crystalline structures of the prepared samples were identified by a Bruker-AXS system (Bruker Company, Karlsruhe, Germany) with Cu-Ka radiation for X-ray diffraction analysis (XRD). An electron probe micro analyzer JED 2300 (JEOL Company, Tokyo, Japan) was used for detecting the elements in the prepared samples through energy dispersive X-ray spectroscopy (EDX). For studying the thermal behavior of the prepared samples, thermogravimetric analyzer TA series Q500 and differential scanning calorimetry (DSC) TA series Q600 (TA company, New Castle, PA, USA) were used under flow of nitrogen. FTIR spectroscopy was performed by using a Perkin–Elmer Spectrum 400 instrument as KBr discs in the range of 425–4000 cm^−1^. For imaging the nano size and morphology of the prepared materials, transmission electron microscopy (TEM) JEM 2100F (JEOL Company, Tokyo, Japan) has used with different magnifications. The optical properties were measured for the prepared samples through the diffuse reflectance technique. UV/VIS/NIR Shimadzu 3600 spectrophotometer (Shimadzu, Columbia, MD, USA) has used for measuring the absorbance of liquid and solid samples.

### 4.5. Photocatalytic Activity

Photocatalytic degradation of aqueous solutions of industrial dyes has used for measuring the photocatalytic activity of the prepared materials for purification of water in sunlight. The photo activities of the prepared nanomaterials were studied through photocatalytic reactions of the green dyes such as Acid green 1 (AG1) in the sunlight. In the current study, 0.1 g of the prepared nanomaterial is well-dispersed in 20 mL of the aqueous solution of AG1 (4 × 10^−4^ M) and exposed to sunlight. Glass test tube was used as a vessel reaction. By measuring the dimensions of the reaction tube, the irradiation area was 10 cm^2^. Depending on the law of Beer-Lambert, the concentration of the dye is proportional to the intensity of the measured spectrum of the dye when the initial concentration is low. By withdrawing a certain amount of the mixture after few minutes of irradiation in sunlight, the absorbance of the diluted (typically 1:2 in water) samples was measured using UV-Vis spectrophotometer. The extent of decomposition of the green dyes is determined by calculating the integrated area of the characteristic peak of AG1 at 714 nm. All photocatalytic degradation experiments were performed under irradiation of sunlight in the period of 10:00 a.m. and 10:20 a.m. during the spring season (March) in Saudi Arabia. The intensity of sunlight was 25 W cm^−2^. With noting that all experiments were performed in sunlight after keeping the sample in dark for 10 min to exclude the adsorption process of the dyes from the calculation.

## 5. Conclusions

In the present study, a dual-aim was achieved for designing zinc oxide nanohybrid to be useful and effective for purifying water in sunlight. This aim focused on a new strategy for building inorganic-magnetic-organic and inorganic-magnetic nanohybrids in addition to producing effective zinc oxide nanohybrid in sunlight. In this line, two different nanohybrids were prepared with and without organic species. The first nanohybrid was produced by combining the nanoparticles of cobalt iron oxides nanocomposites with the nanolayered structures of Al/Zn. The second inorganic-magnetic-organic nanohybrid was formed through expanding the nanolayered structures of Al/Zn by intercalating long chains of hydrocarbons of fatty acids such as stearic acid to facilitate the insertion of very fine nanoparticles of cobalt iron oxides among the nanolayers of Al/Zn. The characterization techniques showed that the prepared nanohybrid were useful for producing zinc oxide nanohybrid by thermal treatment. By measuring the optical properties, a clear reduction of the band gap energy was observed for the prepared zinc oxide nanohybrids comparing with the doped and un-doped zinc oxide. This reduction of the band gap energy from 3.20 eV to 2.75 eV led to high activity for the prepared zinc oxide nanohybrid in sunlight.

This high activity was proven by a complete removal of Acid green 1 after 10 min of sunlight exposure in presence of the prepared zinc oxide nanohybrid. These results were confirmed by the comparison with the pure and doped zinc oxide which indicated that the pure and doped zinc oxide removed the green dyes after 360–840 min of sunlight exposure. In addition, the kinetic study showed that the zinc oxide nanohybrid, which based on inorganic-magnetic-organic nanohybrid, is better than the zinc oxide nanohybrid which produced from inorganic-magnetic nanohybrid. Finally, it can be concluded that this strategy for designing photo-active nanohybrid led to positive tools for facing the energy and water related problems through using renewable and non-polluting energy for purifying water.

## Data Availability

Data available in a publicly accessible repository.

## Sample Availability

Samples of the compounds are not available from the authors.
